# Effects of Periodic Fasting on Fatty Liver Index—A Prospective Observational Study

**DOI:** 10.3390/nu11112601

**Published:** 2019-10-30

**Authors:** Stefan Drinda, Franziska Grundler, Thomas Neumann, Thomas Lehmann, Nico Steckhan, Andreas Michalsen, Francoise Wilhelmi de Toledo

**Affiliations:** 1 Buchinger Wilhelmi Clinic, 88662 Überlingen, Germany; franziska.grundler@buchinger-wilhelmi.com (F.G.); francoise.wilhelmi@buchinger-wilhelmi.com (F.W.d.T.); 2 Clinic St. Katharinental, Department of Rheumatology, 8253 Diessenhofen, Switzerland; 3 Charité–Universitätsmedizin Berlin, Corporate Member of Freie Universität Berlin, Humboldt–Universität zu Berlin and Berlin Institute of Health, 10117 Berlin, Germany; nico.steckhan@charite.de; 4 Division of Rheumatology, Immunology and Rehabilitation, Kantonsspital St. Gallen, 9007 St. Gallen, Switzerland; thomas.neumann@kssg.ch; 5 Institute of Medical Statistics, Computer Sciences and Documentation, Jena University Hospital, 07743 Jena, Germany; thomas.lehmann@med.uni-jena.de; 6 Department of Internal and Complementary Medicine, Immanuel Hospital Berlin, 14109 Berlin, Germany; andreas.michalsen@immanuel.de

**Keywords:** periodic fasting, obesity, overweight, fatty liver index, diabetes

## Abstract

This prospective observational trial investigated effects and safety of periodic fasting in subjects with and without type 2 diabetes mellitus (T2DM). The primary end point was set as the change of fatty liver index (FLI) as a surrogate parameter of non-alcoholic fatty liver disease (NAFLD). Six-hundred and ninety-seven subjects (38 with T2DM) were enrolled. A baseline FLI ≥ 60 (the threshold for fatty liver) was found in 264 subjects (37.9%). The mean duration of fasting was 8.5 ± 4.0 days (range 6–38). FLI decreased significantly (−14.02 ± 11.67; *p* < 0.0001), with a larger effect in individuals with T2DM (−19.15 ± 11.0; *p* < 0.0001; *p* = 0.002 compared to non-diabetic subjects). Body mass index (BMI) decreased by −1.51 ± 0.82 kg/m^2^, and 49.9% of the subjects lost ≥5% body weight. After fasting, nearly half of the 264 subjects with FLI ≥ 60 (highest risk category) shifted to a lower category. The improvement of FLI correlated with the number of fasting days (r = −0.20, *p* < 0.0001) and with the magnitude of BMI reduction (r = 0.14, *p* = 0.0001). Periodic fasting with concomitant weight reduction leads to significant rapid improvement of FLI in subjects with and without T2DM.

## 1. Introduction

Non-alcoholic fatty liver disease (NAFLD) is considered one of the most relevant causes of chronic liver disorders [1], and consists of a disease spectrum including fatty liver, non-alcoholic steatohepatitis, fibrosis, and liver cirrhosis. The least advanced stage of disease, the non-alcoholic fatty liver (NAFL, or simple steatosis), is characterized by an excess of fat in the liver and is mostly asymptomatic [2]. The median prevalence of NAFLD is 20%, ranging from 6% to 33% in industrialized countries [3]. The burden of disease is rapidly increasing, mainly due to the rising prevalence of obesity and sedentary lifestyle, considering that as many as 50% of patients with simple steatosis proceed to develop a non-alcoholic steatohepatitis (NASH) [4].

NAFLD fuels in a closed loop the epidemics of type 2 diabetes mellitus (T2DM) and metabolic syndrome [5], almost doubling the risk of developing these disorders [6]. Notably, NAFLD is regarded as an even stronger predictor for the development of T2DM then waist circumference or obesity [7], and the degree of histologic liver damage is directly related to the presence of diabetes in morbidly obese patients [8]. 

NAFLD is a multi-factorial disease resulting from a complex interaction of environmental and genetic factors. The most common cause of NAFLD is an altered whole-body energetic homeostasis resulting from a caloric intake exceeding the caloric expenditure. Energy spill-over is then stored in the form of non-esterified fatty acids (NEFA) from visceral adipose tissue into ectopic fat depots, such as the liver, skeletal muscles, and pancreas [9]. NAFLD has been associated with dietary excess of saturated fatty acids, refined carbohydrates, and fructose [10,11].

The amount of liver fat can be determined by magnetic resonance imaging (MRI) or biopsy, but it can also be calculated by the fatty liver index (FLI), which has been shown to closely correlate with the results of the MRI [12]. The FLI is calculated on the basis of body mass index (BMI), waist circumference, triglycerides (TG), and gamma-glutamyl-transferase (GGT) [13], a calculation that permits an easy detection of NAFLD and also allows a non-instrumental monitoring of treatment effects. The FLI score ranges from 0 to 100, with FLI <30 excluding and ≥60 confirming a diagnosis of fatty liver (including NAFLD).

Several therapeutic strategies can reverse NAFLD [14], but the most important approach is lifestyle modification with diet and exercise [15,16]. A body weight reduction of 7–10% obtained with energy restriction and increased physical activity leads to histological improvement of steatosis, inflammation, and fibrosis [17,18]. Pharmaceutical approaches, in turn, focus directly on hepatic fat accumulation, anti–inflammatory effects (e.g., oxidative stress alleviation and modulation of tumor necrosis factor), insulin sensitization, or anti–obesity drugs [14]. Thus far, however, these approaches have shown rather limited effects on the progression of fatty liver [16,19,20].

While the combination of physical exercise and change of dietary habits is the most effective intervention for weight loss [21], the translation of long-term lifestyle changes into daily routine seems to be difficult to achieve [22]. One of the proposed options to ease this difficulty is fasting therapy, which is the voluntary renouncement of food for a defined period and which results in distinct metabolic changes, i.e., pronounced lipolysis, decreased insulin secretion, increased insulin sensitivity, gluconeogenesis, and production of ketone bodies [23]. Periodic fasting, in contrast to intermittent or Ramadan fasting, is defined as lasting from 2 to 21 days or longer. Periodic fasting has been used since decades as a treatment option of the metabolic syndrome [23]. Additionally, periodic fasting does not only induce beneficial effects on lifestyle modification, with a subsequent better adherence to nutritional recommendations and exercise [24], but can also counteract a prediabetes by restoring beta cell function and overcoming insulin resistance [25].

To the best of our knowledge, there are no clinical data on the impact of fasting therapy on fatty liver, although beneficial effects of periodic and intermittent fasting on fatty liver have already been shown in mice experiments [26]. Currently, it is also unknown if the effects of fasting on fatty liver are similar in non-diabetic and diabetic individuals. The present study was thus performed to examine the short-term effects of fasting therapy on FLI in diabetic and non-diabetic subjects, and to investigate the safety of this approach.

## 2. Materials and Methods

### 2.1. Study Design

This study was conducted according to the Declaration of Helsinki and was registered at the German register of clinical trials (DRKS-ID: 00010111). The study protocol was approved by the ethics committees of the Charité Medical University of Berlin and the Federal Medical Council of Baden-Württemberg, Germany. 

The subjects agreed to study participation by signing a written informed consent. 

The study was designed as a prospective, uncontrolled, observational cohort study. All subjects were voluntary inpatients at the Buchinger-Wilhelmi Clinic Überlingen (Germany), a medical center specialized on periodic fasting. The data presented in this study are part of a larger trial aimed at investigating safety, health improvement, and well-being in 1422 individuals undergoing periodic fasting [27].

### 2.2. Subjects

Study subjects were consecutively recruited between January and October 2016. Inclusion criteria were age ≥18 years; BMI ≥19 kg/m^2^, and a minimum scheduled inpatient stay of 10 days with at least 6 days for periodic fasting. Exclusion criteria were viral or autoimmune liver diseases, cognitive and psychological disorders, pregnancy or lactation, and inadequate language skills to communicate in English, French, or German.

### 2.3. Study Interventions

All participants underwent periodic fasting according to the published guidelines for periodic fasting therapy [28]. 

The intervention started with a low-calorie transition day (600 kcal/day mono-diet consisting of fruits, rice, oat, or vegetables according to patients’ choice). During the fasting period, the subjects received 250 mL fruit juice or vegetable broth at midday, 250 mL vegetable broth in the evening, and optional 20 g honey (maximum total energy intake of 250 kcal/day; maximum total carbohydrate intake 35 g/day). It was recommended to drink at least 2 liters of water/day. The duration of fasting was adapted to the individual therapeutic goal and tolerance, and was then followed by stepwise reintroduction of food. The latter consisted of ovo-lacto-vegetarian food increasing from 800 kcal/day to 1800 kcal/day over at least 3 days. The fasting therapy was accompanied by a physical exercise program with moderate walking and gymnastics as group activities, paralleled by group sessions in mindfulness, and relaxation techniques as autogenic training and meditation.

### 2.4. Outcome Measures

All measurements were done at baseline (day before start of fasting) and at the end of therapy (day after last fasting day). Anthropometrical measurements (height, weight, and waist circumference) and blood samples for laboratory assessments were taken between 7.00 and 10.00 a.m. Glucose was measured using the hexokinase 3 method (Siemens, Erlangen, Germany; coefficients of variation (CV) 11%; normal range 3.9–5.5 mmoL/L); HbA1c by high pressure liquid chromatography (HPLC; Tosoh Bioscience, Griesheim, Germany, CV 10%, normal <42 mmoL/moL); total cholesterol (TC) by enzymatic color reaction (Siemens; CV 7%; normal <5.2 mmoL/L); triglycerides (TG) by glycerol phosphate oxidase (GPO)/Trinder enzymatic color reaction (Siemens; CV 9%; normal <1.7 mmoL/L); high density lipoprotein (HDL) by a two step catalase–elimination reaction (Siemens; CV 9%; range 1.2–3.1 mmoL/L); and low density lipoprotein (LDL) by a two–step catalase–elimination reaction (Siemens; CV 6.2%; normal <4.1 mmoL/L). Glutamate–oxalacetate transferase (GOT), glutamate–pyruvate transferase (GPT), alkaline phosphatase (AP), and gamma glutamyl transferase (GGT) were measured by standard methods. 

The FLI was calculated as follows: FLI = (e 0.953 × loge (TG) + 0.139 × BMI + 0.718 × loge (GGT) + 0.053 × WC − 15.745)/(1 + e 0.953 × loge (TG) + 0.139 × BMI + 0.718 × loge (GGT) + 0.053 × WC − 15.745) × 100 [13].

The study data were analyzed as per protocol analysis.

### 2.5. Statistical Analysis

The data were presented as means ± standard deviation (SD), if not otherwise indicated. D’Agostino and Pearson omnibus normality test was used to verify the normal data distribution. Changes between baseline and end of intervention were compared by the paired test for normally distributed data and the Wilcoxon matched-pairs signed rank test for non-parametric data. To determine possible differential effects in diabetic patients, all participants (group all) were clustered in non-diabetic subjects (fasting glucose <7 mmoL /L and HbA1c <47 mmoL/moL) and patients with T2DM (fasting glucose ≥7 and/or HbA1c ≥47 mmoL/moL). Group differences were calculated with the unpaired test for parametric data or Mann Whitney U test for non-parametric data. Correlations analyses were performed by the Pearson or Spearman correlation coefficients, depending on the normal distribution of the data.

A multivariable linear regression model was fitted for FLI after intervention with gender, age, number of fasting days, GOT, GPT, total cholesterol, diabetes, and baseline FLI as independent variables. Estimated regression coefficients with 95% confidence intervals (CI) were calculated to evaluate the influence of the variables.

Additionally, a binary logistic regression model was applied for a positive outcome (defined as FLI ≤60 after intervention) only for the subjects who had a baseline FLI >60. Independent variables were gender, age, number of fasting days, GOT, GPT, total cholesterol, diabetes, and baseline FLI. Receiver-operating curve (ROC) analysis of predicted probabilities was performed, and the area under curve (AUC) with 95% CI was calculated to measure the accuracy of the model. The discrimination of this model was assessed by comparing the predicted probabilities with the binary outcomes (FLI ≤ 60) of each patient in the ROC analysis.

A *p* value < 0.05 was considered statistically significant. Data analyses were performed by using the statistical software SPSS version 24 (IBM) and GraphPad Prism 6 (GraphPad Inc.).

## 3. Results

Of 1500 screened subjects, 741 were included in the study as per inclusion/exclusion criteria. Of these, 44 dropped out due to: periodic fasting days <6 (*n* = 27), recently diagnosed liver disease (*n* = 12), or non-compliance (*n* = 5). Thus, 697 subjects completed the study and were analyzed per protocol. The mean fasting duration was 8.5 ± 4.0 days (range 6–38), with no significant difference (*p* = 0.261) between subjects with T2DM (9.3 ± 4.8 days, range 6–31) or without T2DM (8.4 ± 4.0 days, range 6–38).

Of the 697 study subjects, 38 (5.5%) had T2DM. Of those, 28 were treated with anti–diabetic medications as follows: metformin: *n* = 27, dipeptidyl peptidase-4 (DPP4) inhibitors: *n* = 6, sulfonylurea: *n* = 5, sodium-glucose transport 2 (SGLT-2) inhibitors: *n* = 2, and glitazone: *n* = 1. Three patients received a combination of oral anti-diabetics and insulin therapy. One subject received insulin treatment only. 

Treatment for arterial hypertension was recorded in 122 subjects (17.5%), 99/659 in the non-diabetic subgroup and 23/38 in the T2DM subgroup. Treatment with statins was recorded in 116 subjects (16.6%), 78/659 in the non-diabetic subgroup and 18/38 in the T2DM subgroup.

### 3.1. Baseline Characteristics of Study Participants

Baseline characteristics of all study subjects and categorized for the presence or absence of T2DM are presented in Table 1.

The study population was predominantly female and middle-aged. Patients with T2DM were older than subjects without diabetes (60.92 ± 9.74 years vs. 54.23 ± 13.46 years, *p* = 0.002), and their baseline BMI and waist circumference were significantly higher than in non-diabetic subjects.

The FLI was normal (<30) in 273 subjects (39.2%), intermediate (between ≥30 and <60) in 160 subjects (23.0%), and indicative of fatty liver (≥60) in 264 subjects (37.9%). The subgroup of patients with T2DM (*n* = 38) had a significantly higher FLI than subjects without T2DM (78.36 ± 16.97 vs. 44.92 ± 31.57, *p* < 0.001).

### 3.2. Changes in FLI

**Figure 1:**
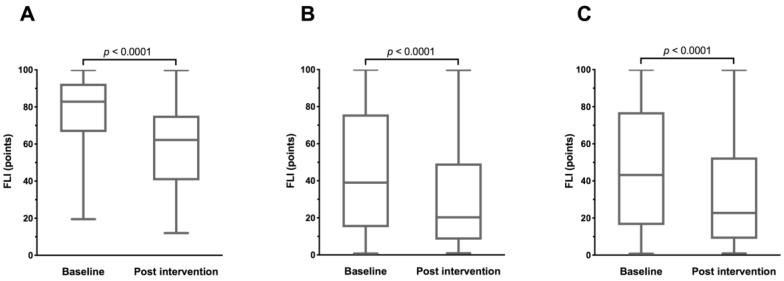
Changes in FLI before and after fasting in patients with T2DM (**A**), non-diabetic subjects (**B**), and all subjects (**C**).

Overall, periodic fasting reduced the FLI by a mean of −14.02 ± 11.67 points (*p* < 0.0001; Figure 1). 

**Figure 2:**
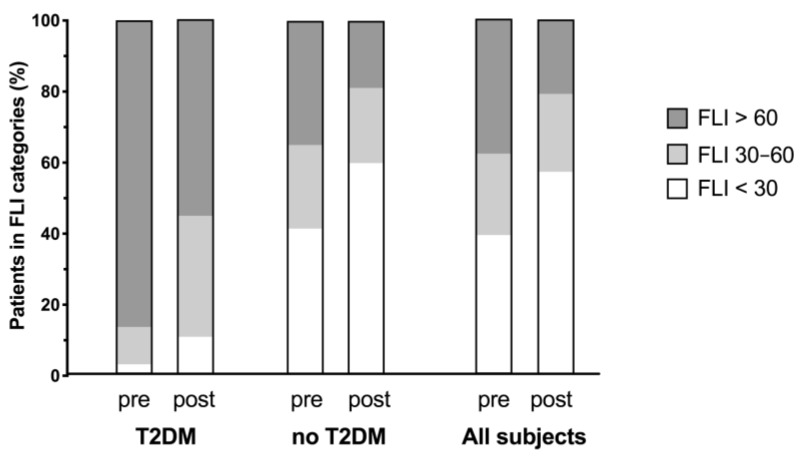
Frequency distribution of FLI categories before and after therapeutic fasting in patients with T2DM (*n* = 38), in non-diabetic subjects (no T2DM; *n* = 659), and in all subjects (*n* = 697).

As many as 120 of the 264 subjects with a baseline FLI ≥ 60 (high risk category) shifted to a lower category of FLI risk after therapy. The subgroup of patients with T2DM (*n* = 38) experienced a significantly greater FLI reduction (−19.15 ± 11.0 points, *p* < 0.0001) than non-diabetic subjects (−13.73 ± 11.65 points, *p* < 0.0001; group difference *p* = 0.002; Figure 2). 

In the non-diabetic subgroup, the number of subjects with fatty liver decreased from 231 to 124 subjects (−46.3%), while the number of subjects with normal FLI (<30 points) increased from 273 to 395 subjects (+44.7%). In the T2DM subgroup, the number of patients with fatty liver decreased from 33 to 21 (−36.4%), while the number of patients with normal FLI increased from 1 to 4. An absolute FLI reduction of >30% was achieved in 60.9% of all subjects who had a weight reduction of ≥5% from baseline.

### 3.3. Changes in Anthropometric and Metabolic Parameters

Changes from baseline are shown in Table 2.

Periodic fasting induced a significant weight loss in the overall population (−4.37 ± 2.42 kg, *p* < 0.001). In the non-diabetic subgroup, the mean changes were −4.31 ± 2.41 kg (*p* < 0.001), in the T2DM subgroup −5.29 ± 2.55 kg (*p* < 0.001). Half of the subjects (348, 49.9%) lost ≥5% of their body weight. Overall, the BMI decreased by −1.51 ± 0.82 kg/m^2^ (*p* < 0.001). In non-diabetic subjects, the BMI decreased by −1.50 ± 0.81 kg/m^2^ (*p* < 0.001), and in T2DM patients by −1.75 ± 0.85 kg/m^2^ (*p* < 0.001).

The waist circumference decreased overall by −5.39 ± 3.28 cm (*p* < 0.001), in non-diabetic subjects by −5.34 ± 3.27 cm (*p* < 0.001), and in T2DM patients by −6.32 ± 3.37 cm (*p* < 0.001). 

Fasting plasma glucose and HbA1c levels significantly decreased in all groups. Liver enzymes also decreased after the fasting intervention, except for AP in patients with T2DM. The same was found for blood lipids. All blood lipids markedly dropped, except for HDL cholesterol and LDL cholesterol in patients with T2DM (Table 2).

### 3.4. Correlations Analyses

The results of the correlation analyses are shown in Table 3.

**Figure 3:**
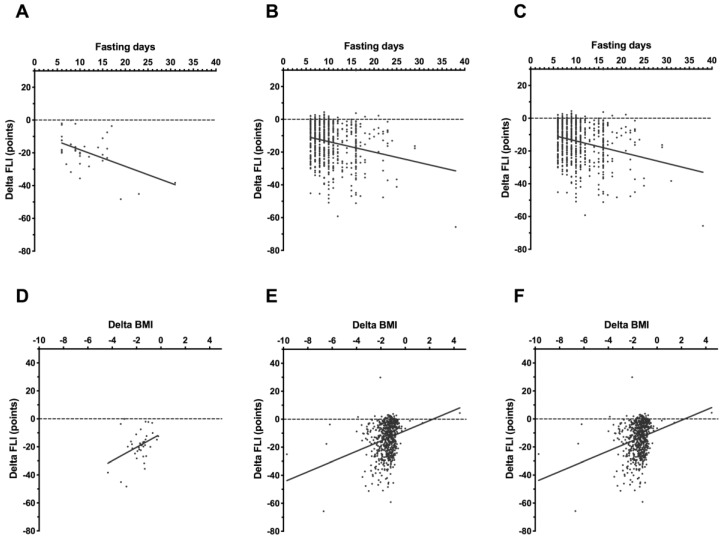
Correlation of FLI changes with fasting duration in patients with T2DM (**A**), non-diabetic subjects (**B**), and all subjects (**C**); and with changes of BMI in patients with T2DM (**D**), non-diabetic subjects (**E**), and all subjects (**F**).

The decreases in FLI induced by fasting correlated with the length of the fasting, in the overall group (r = −0.20; *p* < 0.0001) as well as in the subgroups of non-diabetic subjects (r = −0.18; *p* < 0.0001) and T2DM patients (r = −0.36; *p* = 0.0262; Figure 3).

**Figure 4:**
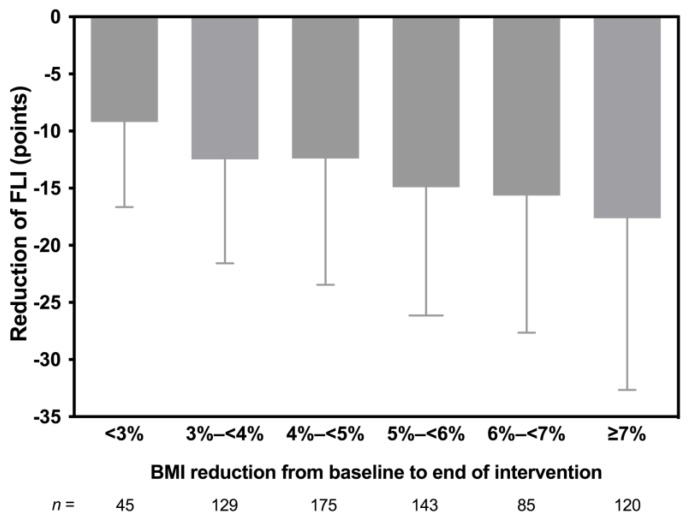
Reduction of FLI in relation to percent changes in body mass index (BMI).

Likewise, changes in FLI significantly correlated with the decrease of body weight and waist circumference (Table 3, Figure 4).

There were no significant correlations between changes in FLI and fasting plasma glucose or HbA1c levels (Table 3).

**Figure 5:**
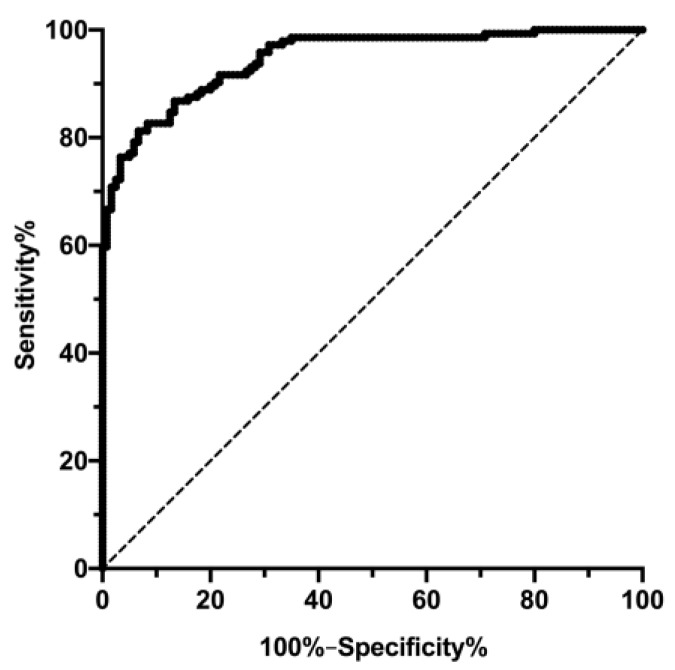
Receiving operator characteristic (ROC) curves of gender, age, number of fasting days, and baseline parameters (GOT, GPT, total cholesterol, diabetes status, and FLI) for the prediction of FLI ≤60 after fasting intervention.

Predictors of changes in FLI were analyzed in a multivariate linear regression model with gender, age, number of fasting days, GOT, GPT, total cholesterol, diabetes, and baseline FLI as independent variables. FLI decreased on average by 0.48 points with each additional fasting day (regression coefficient beta: −0.48, 95% CI −0.665 to −0.295, *p* < 0.001). FLI reduction was also nearly 4 points higher for males (−3.94, 95% CI −5.780 to −2.10, *p* < 0.001) than for females. GOT (10.61, 95% CI 4.77 to 16.46, *p* < 0.001), baseline FLI (−0.139, 95% CI −0.169 to −0.110, *p* < 0.001), and total cholesterol (0.027, 95% CI −0.044 to −0.010, *p* = 0.002) were also significant predictors in this model. A ROC curve was calculated to test the performance of this model (input variables: gender, age, number of fasting days, GOT, GPT, total cholesterol, diabetes, and baseline FLI) in discriminating the capability to shift from a FLI > 60 to a FLI ≤ 60 due to periodic fasting (Figure 5). With this model, the ability to discriminate subjects proved relatively high (AUC = 0.947, 95% CI 0.922–0.971, *p* < 0.001).

### 3.5. Safety

Adverse events were reported in 10 study subjects (1.4%). None of the events met the criteria of seriousness. The following events occurred in more than one subject: eczema (*n* = 3) and mild hyponatremia (*n* = 2). The following events occurred in one subject each: self-limiting supraventricular tachycardia, intermittent self-limiting paroxysmal atrial fibrillation, mild hypokalemia, bleeding gums, common cold, dizziness, mild hypoglycemia, intermittent visual disorders, and headache. None of the subjects discontinued the fasting because of the events.

A daily self-reporting form for feelings of hunger showed that 363 of the 579 respondents (62.7%; 29 of whom with T2DM) did not experience relevant hunger during the fasting therapy, whereas 217 subjects (37.3%) had at least one episode of hunger. No one indicated to be hungry every day during fasting.

## 4. Discussion

Diet interventions are well-established strategies to reduce body weight and improve glucose metabolism, however no evidence exists about the effects of periodic fasting on NAFLD. This study is the first to show the beneficial effects of periodic fasting on fatty liver. The results of our study supported the hypothesis that FLI, a surrogate parameter of fatty liver, significantly decreases in individuals with and without T2DM after a fasting intervention of at least 6 days. The prospective study design according to a standardized protocol with a well-established fasting technique and a close clinical monitoring was a strength of this study.

Weight reduction and improvement of fatty liver indicators are known to be interrelated [17,18]. Our results indicate that even a modest reduction of BMI improved surrogate markers of fatty liver. Indeed, in nearly half of the subjects in the highest risk category (FLI > 60), a BMI reduction of less than 5% was sufficient to induce a shift to a lower risk category.

In most patients, NAFLD is associated with features of metabolic syndrome, central obesity, elevated blood pressure, dyslipidemia, hyperglycemia, and insulin resistance [29]. Although these pathologies can be addressed by lifestyle interventions, in daily life the adherence to the necessary lifestyle changes is poor, resulting in suboptimal outcomes. In contrast, in pragmatic programs there is a greater benefit in a more substantial weight loss, particularly at early stages of the intervention period [30]. Hence, periodic fasting can significantly reduce weight, and this effect can be maintained over time [31]. 

There are several concerns about the adverse effects of fasting. Several non-fatal (e.g., headache) and rarely fatal (e.g., ventricular arrhythmia) events have been reported [32]. In contrast, no severe adverse events were found in a cohort of 1422 subjects treated with a periodic fasting lasting 4–21 days, [27]. Michalsen et al. evaluated the acceptance, safety, effects on health-related outcomes, and lifestyle adherence of fasting therapy in different chronic internal diseases [24]. They found no serious adverse events throughout their study. Our study supports the hypothesis that fasting therapy provided in a controlled clinical setting is a safe intervention.

There is a large body of evidence on the beneficial effects of carbohydrate restriction and hypocaloric diets on NAFLD [33]. It has also been shown that-at equal levels of weight reduction-a carbohydrate-restricted diet leads to a significantly greater intrahepatic triglyceride reduction than low-calorie diet alone [34]. The metabolic advantage of carbohydrate restriction appears to be related to a more pronounced lipid oxidation and enhanced ketogenesis. In recent years, intermittent and periodic fasting has gained popularity as an alternative to continuous caloric restriction. In addition to the weight loss effects, periodic fasting is associated with several metabolic benefits, including the improvement of lipid profiles [27]. This has been also shown for intermittent fasting, e.g., Ramadan fasting [35]. The key mechanism responsible for many of these beneficial effects is the metabolic switch from the utilization of glycogenolysis-derived glucose to fatty acids and fatty acid-derived ketones, i.e., a fundamental switch from lipid synthesis and fat storage to mobilization of fat in the form of free fatty acids and fatty acid-derived ketones. This occurs between 12 and 36 hours after cessation of food consumption [36]. Hyperinsulinemia suppresses ketogenesis and therefore prolongs the time to switch in cases of obesity, insulin resistance, and T2DM [37]. Although there is some evidence of impaired ketogenesis during the progression of liver disease to steatohepatitis, therapies that increase hepatic ketogenesis are expected nonetheless to ameliorate NAFLD [38,39]. 

Our data provided first evidence that periodic fasting leads to a clearance of liver fat: fasting significantly reduced FLI and increased the proportion of patients without NAFLD (FLI < 30 units; Figure 2). The effects of fasting therapy were stronger in males and in individuals with higher baseline FLI, higher GOT, and higher cholesterol levels. Each additional fasting day further decreased the FLI. The binary logistic regression showed that every day of fasting increase by 40% the chance to improve a manifest fatty liver (FLI > 60) and switch to a lower category of risk. This implies that the duration of fasting must be sufficient to influence fatty liver positively. This should be taken into account when periodic fasting is considered as treatment for NAFLD.

As already mentioned, insulin resistance, T2DM, and the development of NAFLD are closely associated conditions. Taylor et al. have shown that remission of T2DM requires a decrease of liver fat [40]. Patients with T2DM tend to have higher FLI scores, but in this study, we could demonstrate that the fasting intervention was equally effective in T2DM patients and in non-diabetic subjects in terms of FLI, although the improvement of other parameters (e.g., HDL, LDL, and AP) was not as complete. These results are in line with a previous study on periodic fasting in T2DM [31].

Liver enzymes, insulin resistance, and cholesterol levels are related in NAFLD [41,42]. Our results supported a correlation between changes in FLI and changes in liver enzymes (GGT, and GOT) and lipid parameters (TG) after fasting intervention, although this was limited to subjects without T2DM.

The intervention was well tolerated and adverse events were rare. Interestingly, the majority of participants did not feel hungry during fasting, as reported also in other studies [32]. Periodic fasting leads to significant weight loss, reduction of BMI and waist circumference. These observations are interpreted as positive effects, considering that at baseline the participants were pre-obese (BMI > 25 kg/m^2^; overall and in the non-diabetic subgroup) or obese (BMI > 30 kg/m^2^; in the T2DM subgroup). Both pre-obesity and obesity are regarded as general health risk factors [43]. 

Our observational study has some limitations. The analyses were carried out as pre- to post intervention changes, without a control group, and were focused on FLI as surrogate parameter for NAFLD, which have been shown to correlate with MRI assessments of fatty liver [12]. MRI assessments are regarded as the gold standard for the diagnostics of NAFLD, but this type of external control was not feasible in this large observational study. There was also no significant difference in fasting duration between diabetic and non-diabetic patients, but we did not match the groups before intervention because of the very different numbers of individuals in each group. Finally, we could not collect data on long-term effects after the fasting intervention, therefore a prediction of sustainability of the fasting effects on hepatic changes is not possible. Lean et al. reported that at 12 months almost half of T2DM patients achieved remission to a no-diabetic state and off antidiabetic drugs after intervention with 3–5 months of formula diet (825–853 kcal/day). It is reasonable to expect that periodic fasting gains similar effects in shorter time with good tolerance [44].

In conclusion, periodic fasting can be regarded as an easily realizable, well-tolerated, non-pharmaceutical intervention, which effectively reduces the FLI. This effect was seen in individuals with and without T2DM. Further studies with a control group and long-term follow-up are needed to better characterize the positive effects of periodic fasting on fatty liver and the adaptations in glucose and lipid metabolism.

## Figures and Tables

**Table 1:** Baseline characteristics, comparisons of subjects with and without type 2 diabetes mellitus (T2DM).

	T2DM	No Diabetes	All	*p* Value T2DM vs. No Diabetes
*n*	38	659	697	
Female, *n* (%)	11 (28.9)	429 (65.1)	440 (63.1)	
Age, years	60.92 ± 9.74	54.23 ± 13.46	54.60 ± 13.36	0.0021
BMI, kg/m^2^	31.79 ± 5.15	27.98 ± 5.25	28.19 ± 5.31	<0.0001
BMI categories <25 kg/m^2^, *n* (%) ≥25 kg/m^2^, *n* (%)	1 (2.6) 37 (97.4)	206 (79.5) 453 (20.5)	207 (29.7) 490 (60.3)	<0.0001
Height, cm	173.21 ± 0.10	169.23 ± 0.09	169.45 ± 0.09	0.0162
Weight, kg	95.45 ± 17.78	80.46 ± 17.89	81.28 ± 18.19	<0.0001
Waist, cm	107.13 ± 11.32	92.06 ± 15.21	92.88 ± 15.40	<0.0001
Glucose, mmoL/L	8.10 ± 2.40	5.19 ± 0.72	5.35 ± 1.11	<0.0001
HbA1c, mmoL/moL	55.2 ± 15.0	35.28 ± 3.94	36.37 ± 6.86	<0.0001
GGT, µkat/L	0.75 ± 0.95	0.47 ± 0.53	0.48 ± 0.57	0.0003
GOT, µkat/L	0.46 ± 0.20	0.40 ± 0.21	0.41 ± 0.21	0.0550
GPT, µkat/L	0.63 ± 0.38	0.49 ± 0.35	0.50 ± 0.35	0.0006
AP, µkat/L	1.06 ± 0.31	1.09 ± 0.30	1.09 ± 0.30	0.5164
Cholesterol, mmoL/L	4.83 ± 1.18	5.60 ± 1.18	5.56 ± 1.19	0.0002
TG, mmoL/L	2.09 ± 0.95	1.52 ± 0.77	1.55 ± 0.80	<0.0001
HDL, mmoL/L	1.17 ± 0.34	1.57 ± 0.47	1.55 ± 0.48	<0.0001
LDL, mmoL/L	3.01 ± 1.02	3.49 ± 1.07	3.46 ± 1.08	0.0083
FLI, points	78.36 ± 16.97	44.92 ± 31.57	46.75 ± 31.86	<0.0001
FLI category <30 points ≥30–<60 points ≥60 points	1 4 33	272 156 231	273 160 264	<0.0001

BMI: body mass index; Waist: abdominal circumference; GGT: gamma glutamyl transferase; GOT: glutamate oxalacetate transferase; GPT: glutamate pyruvate transferase; AP: alkaline phosphatase, Cholesterol: total cholesterol; TG: triglycerides; HDL: high density lipoprotein; LDL: low density lipoprotein. Categorical data were analyzed by Fischer’s exact test (BMI categories) and chi-square test (fatty liver index (FLI) categories).

**Table 2:** Changes from baseline to post fasting, overall and for subjects with and without T2DM.

	T2DM (*n* = 38)		No Diabetes (*n* = 659)		All (*n* = 697)		
	Mean ± SD	*p* Value	Mean ± SD	*p* Value	Mean ± SD	*p* Value	*p* Value T2DM vs. No Diabetes
Weight, kg	−5.29 ± 2.56	<0.0001	−4.31 ± 2.41	<0.0001	−4.37 ± 2.42	<0.0001	0.0045
BMI, kg/m^2^	−1.75 ± 0.85	<0.0001	−1.50 ± 0.81	<0.0001	−1.51 ± 0.82	<0.0001	0.0213
Waist, cm	−6.32 ± 3.37	<0.0001	−5.34 ± 3.27	<0.0001	−5.39 ± 3.28	<0.0001	0.0433
FLI, points	−19.15 ± 11.00	<0.0001	−13.73 ± 11.65	<0.0001	−14.02 ± 11.67	<0.0001	0.0020
Glucose, mmoL/L	−2.69 ± 2.56	<0.0001	−0.60 ± 1.24	<0.0001	−0.72 ± 1.42	<0.0001	<0.0001
HbA1c, mmoL/moL	−4.43 ± 6.65	<0.0001	−1.60 ± 2.91	<0.0001	−1.76 ± 3.28	<0.0001	<0.0001
GGT, µkat/L	−0.18 ± 0.37	<0.0001	−0.12 ± 0.27	<0.0001	−0.12 ± 0.28	<0.0001	0.0694
GOT, µkat/L	0.29 ± 0.30	<0.0001	0.20 ± 0.28	<0.0001	0.21 ± 0.29	<0.0001	0.0613
GPT, µkat/L	0.34 ± 0.45	<0.0001	0.18 ± 0.34	<0.0001	0.18 ± 0.35	<0.0001	0.0147
AP, µkat/L	−0.03 ± 0.19	0.3297	−0.04 ± 0.14	<0.0001	−0.04 ± 0.15	<0.0001	0.9886
Cholesterol, mmoL/L	−0.44 ± 1.05	0.0136	−0.66 ± 0.78	<0.0001	−0.64 ± 0.79	<0.0001	0.1858
TG, mmoL/L	−0.78 ± 0.96	<0.0001	−0.43 ± 0.69	<0.0001	−0.44 ± 0.71	<0.0001	0.0057
HDL, mmoL/L	−0.07 ± 0.27	0.0905	−0.24 ± 0.26	<0.0001	−0.23 ± 0.27	<0.0001	<0.0001
LDL, mmoL/L	−0.20 ± 1.03	0.2327	−0.31 ± 0.79	<0.0001	−0.30 ± 0.81	<0.0001	0.6141

BMI = body mass index; Waist = abdominal circumference; GGT = gamma glutamyl transferase; GOT = glutamate oxalacetate transferase; GPT = glutamate pyruvate transferase; AP = alkaline phosphatase, Cholesterol = total cholesterol; TG = triglycerides; HDL = high density lipoprotein; LDL = low density lipoprotein. Data were analyzed by a paired *t*–test.

**Table 3:** Correlation analyses, overall and for subjects with and without T2DM.

	T2DM (*n* = 38)		No Diabetes (*n* = 659)		All (*n* = 697)	
	r	*p* Value	r	*p* Value	r	*p* Value
FLI vs. Fasting days	−0.42	0.0091	−0.18	<0.0001	−0.20	<0.0001
FLI vs. BMI	0.32	0.0474	0.27	<0.0001	−0.14	0.0001
FLI vs. WC	0.39	0.0165	0.28	<0.0001	0.29	<0.0001
FLI vs. GGT	0.22	0.1907	0.48	<0.0001	0.47	<0.0001
FLI vs. GOT	0.18	0.2716	−0.10	0.0102	−0.10	0.0120
FLI vs. GPT	0.18	0.2802	−0.02	0.6154	−0.02	0.5673
FLI vs. AP	0.35	0.0322	0.17	<0.0001	0.18	<0.0001
FLI vs. Cholesterol	0.33	0.0451	0.30	<0.0001	0.29	<0.0001
FLI vs. TG	0.23	0.1576	0.63	<0.0001	0.62	<0.0001
FLI vs. fG	0.03	0.8563	0.07	0.0942	−0.02	0.6890
FLI vs. HbA1C	0.14	0.4096	0.04	0.3186	0.06	0.0891

BMI = body mass index; WC = waist circumference; GGT = gamma glutamyl transferase; GOT = glutamate oxalacetate transferase; GPT = glutamate pyruvate transferase; AP = alkaline phosphatase, Cholesterol = total cholesterol; TG = triglycerides; fG = fasting glucose. r = Pearson’s correlation coefficient for parametric data or Spearman’s correlation coefficient for non-parametric data.
